# Human Erythrocytes Selectively Bind and Enrich Infectious HIV-1 Virions

**DOI:** 10.1371/journal.pone.0008297

**Published:** 2009-12-14

**Authors:** Zoltan Beck, Bruce K. Brown, Lindsay Wieczorek, Kristina K. Peachman, Gary R. Matyas, Victoria R. Polonis, Mangala Rao, Carl R. Alving

**Affiliations:** 1 Division of Retrovirology, United States Military HIV Research Program, Walter Reed Army Institute of Research, Rockville, Maryland, United States of America; 2 Henry M. Jackson Foundation for the Advancement of Military Medicine, Rockville, Maryland, United States of America; University Hospital Zurich, Switzerland

## Abstract

Although CD4(+) cells represent the major target for HIV infection in blood, claims of complement-independent binding of HIV-1 to erythrocytes and the possible role of Duffy blood group antigen, have generated controversy. To examine the question of binding to erythrocytes, HIV-1 was incubated *in vitro* with erythrocytes from 30 healthy leukapheresis donors, and binding was determined by p24 analysis and adsorption of HIV-1 with reduction of infectivity for CD4(+) target cells. All of the cells, regardless of blood group type, bound HIV-1 p24. A typical preparation of erythrocytes bound <2.4% of the added p24, but erythrocytes selectively removed essentially all of the viral infectivity as determined by decreased infection of CD4(+) target cells; however, cell-associated HIV-1 was approximately 100-fold more efficient, via *trans* infection, than unadsorbed virus for infection of CD4(+) cells. All of the bound HIV-1 p24 was released by treatment of the cells with EDTA, and binding was optimized by adding Ca^2+^ and Mg^2+^ during the washing of erythrocytes containing bound HIV-1. Although the small number of contaminating leukocytes in the erythrocyte preparation also bound HIV-1 p24, there was no significant binding to CD4, and it thus appears that the binding occurred on leukocytes at non-CD4 sites. Furthermore, binding occurred to erythrocyte ghosts from which contaminating leukocytes had been previously removed. The results demonstrate that erythrocytes incubated *in vitro* with HIV-1 differentially adsorb all of the infectious HIV-1 virions (as opposed to non-infectious or degraded virions) in the absence of complement and independent of blood group, and binding is dependent on divalent cations. By analogy with HIV-1 bound to DC-SIGN on dendritic cells, erythrocyte-bound HIV-1 might comprise an important surface reservoir for *trans* infection of permissive cells.

## Introduction

Upon initial entry into blood, HIV-1 is faced with three major options: (a) directly infect a CD4(+) target cell; (b) remain as a circulating free virion while it searches for a target cell; or (c) temporarily bind to the surface of a CD4(−) cell, such as a circulating dendritic cell, as a depot for infection by transfer of the virus (infection *in trans*) to a CD4(+) cell [1]. Infection of a CD4(+) target cell is the goal to which the other two options are directed, but it is also the most difficult to achieve because CD4(+) cells are relatively rare when compared to other circulating cells in the blood. Although free infectious HIV-1 is widely thought to be a major form of the virus in blood [1], remaining as a free virion is also fraught with danger because of exposure to numerous humoral and cellular defense mechanisms, such as antibodies, complement, or NK cells available to the host [2]–[6]. Calcium-dependent binding of HIV-1 to DC-SIGN on dendritic cells can also occur, and HIV-1 attached to dendritic cells might be protected for more than 4 days either from degradation or neutralization by certain antibodies, when compared to free HIV-1 [7], [8]. Although *trans* infection of CD4(+) cells by HIV-1-bound dendritic cells has also been proposed based on *in vitro* experiments, direct *in trans* infection has not yet been demonstrated to occur *in vivo* [9], and dendritic cells are relatively rare when compared to other circulating blood cells.

Claims by several groups that HIV-1 also binds to erythrocytes [10]–[13] have generated controversy. Erythrocytes derived from infected patients were reported to contain cell-bound HIV-1, and erythrocytes were hypothesized to represent a novel viral reservoir [12], [14], but erythrocyte-bound HIV-1 from infected patients was subsequently disputed and the reservoir hypothesis was challenged [15]. It was subsequently concluded that erythrocytic complement receptor type 1 (CR1) was responsible for essentially all of the binding of HIV-1 to the erythrocytes [16]. Binding to the erythrocytic blood group Duffy antigen receptor for chemokines (DARC) was also reported [10], [13], but claims that DARC mediated genetic tendencies to HIV-1 infection and AIDS [13], [17]–[19] were strongly challenged [20]–[23].

If erythrocytes are major, or even minor, HIV-1 binding sites not mediated by effectors such as antibodies or complement, this could represent a novel mechanism for transmission of infectious HIV-1 virions to CD4(+) cells in blood. If HIV-1 bound to erythrocytes represents a hidden infectious depot of HIV-1 *in vivo*, this might also present new challenges for development of broadly neutralizing antibodies for a vaccine. In view of the importance and the controversial nature of the issue of HIV-1 binding to erythrocytes, we examined the binding of HIV-1 both to human erythrocytes and to highly purified erythrocytic ghosts in order to ascertain the degree, if any, that infectious HIV-1 virions bind to erythrocytes in the absence of antibodies or complement, and to shed light on possible implications of such binding for concepts of HIV-1 pathogenesis and mechanisms of transmission for uptake and infection of CD4(+) cells. Our results demonstrate that although a mean of only 2.3% of added HIV-1 p24 became bound to erythrocytes, adsorption with the cells differentially removed nearly all of the infectious virions. The erythrocyte-bound HIV-1 was then approx 100-fold more infectious, via *trans* infection, for infection of CD4(+) target cells, and the cell-bound HIV-1 reconstituted essentially all of the infectivity of the original unadsorbed free virus.

## Results

### Binding of HIV-1 to erythrocytes obtained after leukapheresis

**Figure 1:**
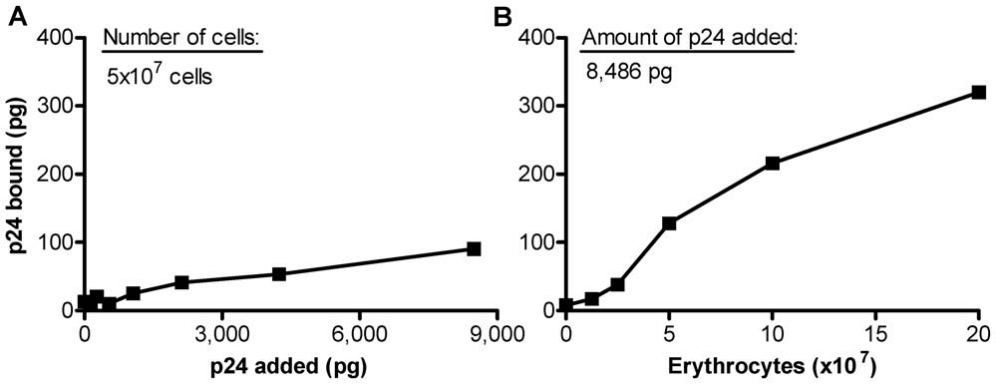
Binding of a HIV-1 isolate to erythrocytes. (A) Increasing amounts of HIV-1 isolate 90US_873 (as quantified by p24) were incubated with 5×10^7^ erythrocytes (donor Q6), and binding of p24 to the cells was determined. (B) Dose-dependent binding of the HIV-1 isolate (8,486 pg p24) with increasing numbers of erythrocytes. The experiment shown is representative of 3 separate experiments. In each experiment HIV-1 was bound to erythrocytes in triplicate, washed, and the triplicates were pooled for p24 determination.

**Figure 2:**
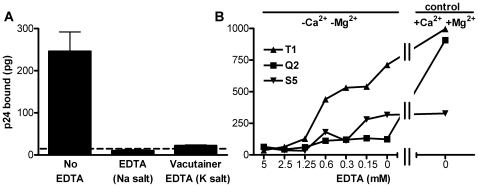
Release of erythrocyte-bound HIV-1 by treatment with EDTA. (A) HIV-1 isolate 89BZ_167 (75,643 pg) was incubated with 5×10^8^ erythrocytes (donor S4) in RPMI, washed three times, and then treated with either EDTA or RPMI (no EDTA). The bound HIV-1 was completely removed by treatment with EDTA. The dashed line shows the limit of detection of the p24 assay. (B) After incubation of HIV-1 with erythrocytes from 3 different donors, following washing, the cells were incubated with different amounts of EDTA (or no EDTA), and washed either in the absence or presence (control) of Ca^2+^ and Mg^2+^.

After incubation of increasing concentrations of a typical preparation of erythrocytes with HIV-1, followed by washing of the cells, dose-related binding of HIV-1 p24 was observed (Fig. 1A). At the highest concentration (8,486 pg of p24, corresponding to a 1∶1 dilution of the viral stock with phenol red-free RPMI), a plateau of binding was still not apparent. However, when 8,486 pg of HIV-1 was then incubated with increasing numbers of erythrocytes, nearly a four-fold increase of p24 binding occurred, resulting in 320 pg of p24 bound per 20×10^7^ erythrocytes (Fig. 1B). Although a definitive plateau of binding was not quite achieved, the change of slope at high numbers of cells indicated that only a very shallow dose response occurred at the high end of the curve. Thus, in the experiment illustrated only 3.7% of the total p24 added became bound to the cells. Erythrocyte preparations obtained from 30 different donors bound a mean of 2.32% (range 0.03–6.02%), of added p24 of undiluted virus stock incubated with the indicated number of erythrocytes (Table 1). Within this small range of binding, the ratio of added cells/viral p24 bore little exact resemblance to the % of p24 bound with different donor cells. Although erythrocytes from each donor preparation did bind HIV-1, the number of individual samples tested was too small to determine contributing effects, if any, of each potential variable (such as blood group type, or viral clade, or type of co-receptor used by the virus) shown in Table 1. Although the exact mechanism of binding of the HIV-1 virions to the cells is not yet known, Fig. 2A demonstrates that the binding was completely eliminated in the presence of EDTA. As shown with three representative donor preparations in Fig. 2B, removal of HIV-1 bound to the cells was dependent on the concentration of EDTA. Even in the absence of EDTA, binding of p24 to two of the three donor cells was considerably reduced when the medium used to wash the cells lacked Ca^2+^ and Mg^2+^ when compared to the control in which the cells were washed in the presence of Ca^2+^ and Mg^2+^ (Fig. 2B).

**Table 1:** Binding of four HIV-1 isolates to erythrocytes derived from 30 different donors.

Donor no.	ABO blood type	Rh	Duffy blood type	Virus isolate	Viral clade/co-receptor	p24 added (pg)	No. of erythrocytes added	% of p24 bound to cells^a^
Q1	AB	+		99KE_KNH1135	A/R5	5,999	5×10^7^	4.24^b^
Q2	A	+		89BZ_167	B/R4	30,257	5×10^8^	3.48^c^
Q3	B	+		89BZ_167	B/R4	30,257	5×10^8^	1.09
Q4	O	+		89BZ_167	B/R4	12,053	5×10^8^	1.06
Q5	O	+	Fy(a+b-)	89BZ_167	B/R4	30,257	5×10^8^	2.30^d^
				91US_4	B/R5	77,236	5×10^8^	0.03
				99KE_KNH1135	A/R5	29,995	5×10^8^	0.61
Q6	O	+	Fy(a+b+)	99KE_KNH1135	A/R5	29,995	5×10^8^	0.53
				91US_4	B/R5	77,236	5×10^8^	0.28
				90US_873	B/R5	8,486	2×10^8^	3.77
				89BZ_167	B/R4	30,257	5×10^8^	2.66^b^
Q7	O	+	Fy(a+b+)	99KE_KNH1135	A/R5	29,995	5×10^8^	1.23
				91US_4	B/R5	77,236	5×10^8^	0.66
Q8	O	−	Fy(a+b−)	91US_4	B/R5	77,236	5×10^8^	0.36
				99KE_KNH1135	A/R5	29,995	5×10^8^	1.41
				90US_873	B/R5	8,486	2×10^8^	3.65
Q9	A	+	Fy(a−b−)	90US_873	B/R5	8,486	5×10^8^	1.35
				99KE_KNH1135	A/R5	29,995	5×10^8^	0.88
				91US_4	B/R5	77,236	5×10^8^	0.18
				89BZ_167	B/R4	30,257	5×10^8^	2.99
R1	O	−		89BZ_167	B/R4	30,257	5×10^8^	5.61
R2	A	+		89BZ_167	B/R4	15,129	5×10^8^	2.49^b^
R3	O	−		89BZ_167	B/R4	45,386	7.5×10^8^	4.47^b^
R4	O	+		89BZ_167	B/R4	30,257	5×10^8^	2.91
R5	O	+		89BZ_167	B/R4	30,257	5×10^8^	4.37
R6	O	+		89BZ_167	B/R4	30,257	5×10^8^	1.84
R7	A	+		89BZ_167	B/R4	30,257	5×10^8^	6.02
R8	O	+		89BZ_167	B/R4	30,257	5×10^8^	5.67
R9	O	+		89BZ_167	B/R4	7,564	2.5×10^8^	1.07
S1	A	+		89BZ_167	B/R4	15,129	5×10^8^	1.59
S2	O	+		89BZ_167	B/R4	45,386	7.5×10^8^	0.69
S3	A	−		89BZ_167	B/R4	30,257	5×10^8^	3.27
S4	O	+		89BZ_167	B/R4	15,129	5×10^8^	5.40
S5	O	+		89BZ_167	B/R4	72,320	3×10^9^	0.56
S6	A	+		89BZ_167	B/R4	15,129	5×10^8^	4.40
S7	A	+		89BZ_167	B/R4	15,129	5×10^8^	5.55
S8	O	+		89BZ_167	B/R4	7,564	5×10^8^	0.75
S9	B	+		89BZ_167	B/R4	7,564	5×10^8^	0.49
T1	O	+		89BZ_167	B/R4	72,320	3×10^9^	1.65
T2	A	+		89BZ_167	B/R4	72,320	3×10^9^	3.00
T3	A	+		89BZ_167	B/R4	72,320	3×10^9^	0.67
							Mean	2.32
							SD	1.82
							Median	1.65
							Range	0.03–6.02

a Values are from a single experiment except for means of ^b^two, ^c^four or ^d^five experiments.

### Binding of HIV-1 to contaminating leukocytes in the erythrocyte preparation, and binding to erythrocyte ghosts

**Figure 3:**
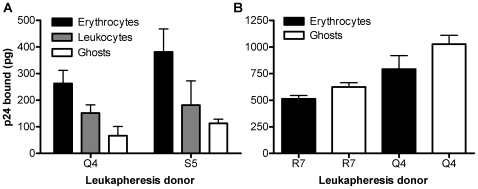
HIV-1 binds to erythrocytes, erythrocytic ghosts, and leukocytes. (A) After incubation of 151,286 pg of HIV isolate 89BZ_167 with the indicated preparation containing 2.5×10^9^ erythrocytes, the mean p24 bound (± SD of triplicate measurements) was determined. The erythrocytes were then hemolyzed and the p24 bound to the ghosts and the leukocytes was separately measured. (B) After incubation of 105,900 pg of HIV-1 isolate 89BZ_167 with the indicated erythrocyte preparation (3.5×10^9^ erythrocytes), or with purified ghosts previously depleted of leukocytes, from each donor, the mean bound p24 (± SD of triplicate measurements) was determined.

Erythrocytes obtained after leukapheresis would still be expected to have a small contamination with leukocytes, including CD4(+) cells. Flow cytometry analysis of one erythrocyte preparation (not shown) revealed approximately 0.4% contamination by non-hemolyzed cells, and 0.07% of the original starting cells in the erythrocyte preparation consisted of CD4(+) cells while 0.33% consisted of CD4(−) cells. To examine the possibility that contaminating leukocytes might have contributed to HIV-1 binding, we hemolyzed the erythrocytes and separated the leukocytes by low speed centrifugation from the erythrocytic ghosts. As shown in Fig. 3A, the non-hemolyzed cells (leukocytes), and the purified erythrocytic ghosts obtained by high speed centrifugation, each bound p24. Based on the smaller numbers of leukocytes, it appears that the binding to leukocytes was stronger than binding to erythrocytes. However, Fig. 3B further demonstrates that HIV-1 also bound directly to purified ghosts from which all contaminating leukocytes had been previously removed. Under these latter circumstances binding to erythrocytic ghosts occurred at approximately the same level as to the original erythrocyte preparation that included contaminating leukocytes. The increased binding to erythrocytic ghosts in the absence of leukocytes (Figs. 3B vs. 3A) suggests that adhesion molecules present on erythrocytes [24] might have competed with adhesion molecules on leukocytes for binding to HIV-1. An alternative possibility that intracellular surface binding sites for HIV-1 became available on ghosts that were stabilized after hemolysis seems unlikely because of limited access to such sites [25].

### Lack of significant binding to contaminating CD4(+) cells in the erythrocyte preparation

**Figure 4:**
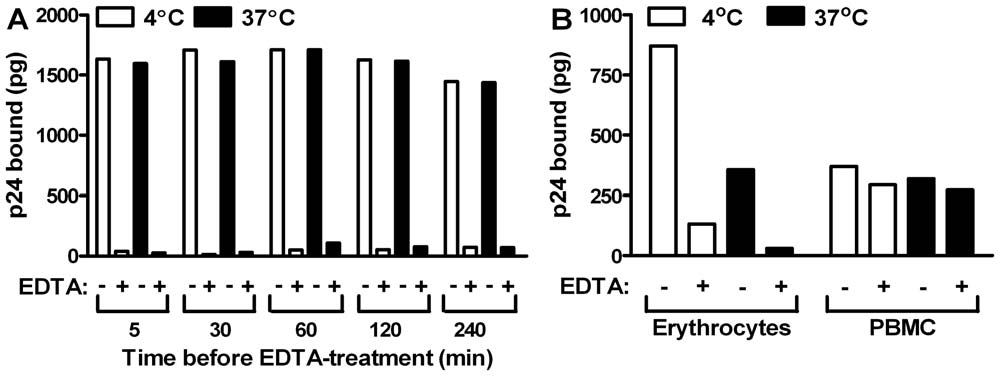
HIV-1 bound to the erythrocyte cell preparation is not internalized by contaminating leukocytes at 37°C. (A) Erythrocytes (donor R9) were first incubated with HIV-1 (isolate 89BZ_167) at 4°C for 2 hr and binding was determined. The cells were then incubated further at either 4°C or 37°C, and at the indicated times 10 ml of 5 mM EDTA (Na salt) were added and p24 associated with the cells was measured. The experiment shown was performed twice with similar results. (B) In a further experiment, containing a positive control, erythrocytes (donor T4) and PBMC were first incubated with HIV-1 (isolate 89BZ_167) either at 4°C or 37°C for 2 hr and binding was determined. The cells were then incubated for 4 hours at either 4°C or 37°C then treated with 5 mM EDTA (Na salt). Cells were washed and p24 associated with the cells was measured.

Because of the binding of HIV-1 to contaminating leukocytes (Fig. 3A), the question arose whether the apparent binding of HIV-1 to non-hemolyzed cells actually consisted of binding to the small number of contaminating CD4(+) cells. If this were so, it would be expected that incubation of the cells at 37°C for 4 hours would allow sufficient time for internalization of the virus [26], and the association of the HIV-1 p24 with the cells would then no longer be susceptible to EDTA. To examine this, HIV-1 was incubated with the cell preparation for 2 hr at 4°C and EDTA was then added at different times over a period of 4 hours during further incubation at 37°C compared to 4°C. As shown in Fig. 4A, at each time interval in the course of the incubation, HIV-1 p24 binding was removed from the cells by EDTA to an equal extent at 37°C and 4°C. In contrast, Fig. 4B shows a partial repeat of the experiment shown in Fig 4A that also contains a positive control performed with peripheral blood mononuclear cells (PBMC) instead of erythrocytes. In the positive control, the PBMC were first incubated with virus at 4°C; after removal of unbound virus by washing, the PBMC were incubated at 37°C for 4 hours and then treated with EDTA. As shown, the p24 associated with PBMC was not removed by treatment with EDTA, thus indicating that the p24 became internalized by PBMC, and because p24 was not on the cell surface it could not be removed from the PBMC by EDTA. Thus, the binding of HIV-1 p24 to erythrocytes occurred mainly, or exclusively, on the surface of the cells, and binding of the virus to CD4 sites on contaminating CD4(+) cells did not play a significant role. Based on this, it appears that the binding of p24 to leukocytes (shown in Fig. 3) did not involve binding of infectious virions to CD4.

### Binding of infectious HIV-1 virions by erythrocytes, and infectivity of bound virus

**Figure 5:**
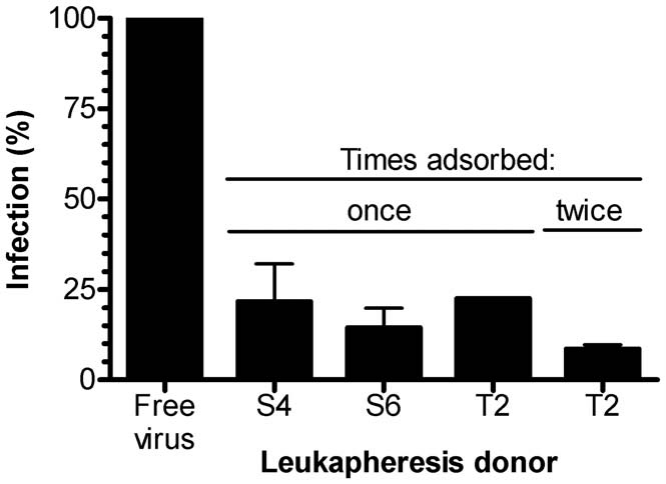
Erythrocyte preparations selectively adsorb most of the infectious HIV-1 virions. HIV-1 isolate 89BZ_167 (105,900 pg) was adsorbed with erythrocytes the indicated numbers of times by adding 3.5×10^9^ erythrocytes from the indicated donors. After removing the erythrocytes by centrifugation, non-adsorbed virus in the supernatant was assayed for the ability to infect CD4(+) TZM-bl cells *in trans*. The relative degree of infection, determined by the mean p24 (± SD of triplicate measurements), was compared with infection by the original free virus (shown as 100%).

**Figure 6:**
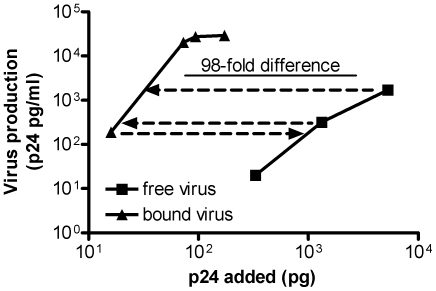
Erythrocyte-adsorbed virus is more infectious, based on p24, than free virus. To compare the infectivity of cell-bound virus with free virus-1, HIV isolate 90US_873 was bound to erythrocytes (sample no. Q6). Increasing numbers of erythrocytes containing bound virus were then added to a PBMC culture, and virus production was determined on day 4 post-infection. The amount of virus production was then compared with that obtained by incubating the original free virus with PBMC. A similar result was obtained in a second experiment with a different donor (no. T1) and HIV-1 isolate 89BZ_167.

Although the above data demonstrated that only a minute fraction of the p24 added became bound to the erythrocytes, Fig. 5 shows that a single adsorption with erythrocytes from each of three different donors depleted an average of approximately 81% of the infectivity of the original HIV-1 preparation, and a second adsorption with one of the erythrocyte preparations removed 92% of the infectivity. In another experiment (not shown) three adsorptions removed 97% of the infectivity. The question then arose whether cell-bound HIV-1 retains its infectivity. Figure 6 shows, in a representative experiment, that when the infectivity of cell-bound HIV-1 was compared to the original unadsorbed free virus, based on p24 values the bound HIV-1 was approximately 100-fold more efficient, via *trans* infection, for infecting CD4(+) peripheral blood mononuclear target cells. It should be emphasized that the 100-fold degree of increased infectivity is only a rough estimate. Even though approximately the same level was seen in two separate experiments, the exact degree of increased infectivity might change under different conditions, and this might also prove to be virus- and cell-dependent. The results demonstrate that erythrocytes bound only an extremely small amount of the total p24, but this accounted for nearly all of the infectious virions, and the cell-bound HIV-1 remained fully infective for transmission to CD4(+) cells by *trans* infection. The data thus show that infectious virions were strongly enriched by binding to the cells, but the data do not exclude the possibility that the cell-bound p24 might also have contained a small fraction of non-infectious particles, such as immature or defective virions, in addition to the infectious particles.

## Discussion

As noted earlier (Introduction), the question of whether HIV-1 binds to erythrocytes, either *in vitro* or *in vivo*, has generated some degree of controversy. In the presence of immune complexes (HIV-1-anti-HIV-1), and in the presence of complement (unheated human serum) *in vitro*, partially purified formaldehyde-inactivated ^125^I-labeled HIV-1 reportedly bound 65–75% of the added viral label to purified erythrocytes from healthy donors after incubation of the labeled HIV-1 with the cells [16]. In contrast, in the absence of complement (using heat-inactivated serum) there was essentially no binding of HIV-1 to the cells. Based on the above, it was concluded that erythrocytic complement receptor type 1 (CR1) was responsible for essentially all of the binding of HIV-1 to the erythrocytes. However, in the present study, which was conducted *in vitro* in the complete absence of antibodies or complement, we demonstrate that while a mean of only 2.3% of HIV-1 p24 bound to erythrocytes, this small amount of binding accounted for binding of up to 97% of the infectious virions, and the cell-bound virions were then approximately 100-fold more infectious, via *trans* infection, for infecting CD4(+) cells. Moreover, the binding occurred even with purified erythrocytic ghosts that were devoid of contaminating leukocytes.

It is known that HIV-1 activates complement, and complement-coated HIV-1 can bind to complement receptors *in vivo*, particularly to CR1 on erythrocytes, or to complement receptor types 2, 3, or 4, in the presence or absence of immune complexes [2], [4], [5], [16], [27], and cell-bound HIV-1 could bind to Fc receptors on CD4(−) cells in the presence of circulating IgG antibodies to HIV-1 [4], [5]. However, the present experiments, performed in the absence of antibodies or complement, suggest that uptake of infectious HIV-1 virions by erythrocytes and other CD4(−) cells by immune adherence to erythrocytic CR1 via complement or complement-mediated immune complexes, or even Fc-mediated adherence to Fc receptors, may not be required for CD4-independent adherence of infectious HIV-1 virions to erythrocytes and other CD4(−) cells. In fact, most of the infectious HIV-1 was differentially adsorbed to cell surfaces through CD4-independent binding in the absence of antibodies. The present results suggest that the small fraction of the total HIV-1 p24, that represents virtually all of the infectious HIV-1 virions, might be bound mainly to erythrocytes and other CD4(−) cells, and that all of the binding occurs at sites other than CR1.

Although the exact mechanism of binding of HIV-1 to erythrocytes is still unknown, and there might be more than one binding mechanism, numerous nonimmune mechanisms potentially could play a role, including binding to cell surface glycolipids [28]–[31], and binding to cell surface glycosaminoglycans and proteoglycans, such as heparan sulfate [32]–[35]. The possibility of binding to HIV-1 to DARC has also been proposed based on *trans* infection mediated by DARC(+) erythrocytes [10], [13]. Although our data do not exclude DARC as one possible erythrocytic binding site, our observation that cells from 30 random plasmapheresis donors, including those with DARC(+) [Fy(a+b+), or Fy(a+b−)] and DARC(−) [Fy(a−b−)] phenotypes, all bound HIV-1 p24 to approximately the same degree (Table 1) makes it seem unlikely that binding to DARC, if it occurs, is the sole binding mechanism. Our current results thus appear to be compatible with the various studies that have challenged binding of HIV-1 to DARC as a critical factor in the epidemiology of HIV-1 infections and pathogenesis [20]–[23].

An interesting aspect of this study is that cell-bound HIV-1 was completely released by EDTA, and binding of HIV-1 therefore occurred only on the cell surface. Even though the infectious HIV-1 particles were completely stripped off cells by treatment with EDTA, because the infectious particles comprised only a small fraction (mean of 2.3%) of the total p24, plasma viral load measurements obtained with calcium chelators such as EDTA as anticoagulants probably detect mainly non-infectious or degraded HIV-1. It thus seems likely that erythrocyte binding might be detected poorly, or not detected, in the presence of any blood collection method that uses calcium binders such EDTA, citrate, or heparin. This might therefore explain the previous inability to detect HIV-1 bound to erythrocytes obtained in EDTA tubes from HIV-1-infected patients [15].

Our observation of highly efficient transfer of virus from CD(-) sites for *trans* infection of permissive CD4(+) cells, is in keeping with other types of assay systems that have suggested that efficient *trans* infection can be experimentally achieved *in vitro* [9], [36]–[38]. However, in view of the possibility of complement-independent and CD4-independent selective binding of virions to erythrocytes, and perhaps to many other cells, with subsequent efficient *trans* infection of permissive cells, the theoretical possibility arises that CD4(−) cell-bound infectious virions might be more important for HIV-1 infection *in vivo* than infection by cell-free virions. If this were so, it would seem to be important to explore whether antibodies that neutralize free virus *in vitro* can similarly neutralize *trans* infection caused by erythrocyte-bound virions. Assays for detection of neutralizing antibodies generally use free virus (or pseudovirus) as the infecting agent [39]. However, as illustrated in the present work, because of the huge number of degraded particles in the free virus preparations commonly used in most neutralizing antibody assays, noninfectious particles or proteins might also serve as a nonproductive sink for the antibodies being tested. It might be further speculated, by analogy with HIV-1 that binds to DC-SIGN on dendritic cells and remains available for more than 4 days for *trans* infection of CD4(+) cells [7], or against neutralization by certain antibodies, when compared to free HIV-1 [8], that infectious HIV-1 bound to the erythrocyte surface *in vivo* might also be present in a type of protected state. We are currently creating a novel *trans* infection neutralization assay to examine the ability of antibodies to neutralize erythrocyte-bound when compared to free HIV-1.

Although it is widely believed that prior to infecting CD4(+) cells, HIV-1 in blood exists mainly as free (i.e., not cell-associated) infectious virions [1], the data from this study supports the hypothesis that the erythrocytes may be added to dendritic cells as a type of cell that binds HIV-1 on the cell surface in a calcium-dependent, CD4-independent, and complement-independent manner. However, erythrocytes might also have the unique capacity to selectively bind all of the infectious HIV-1 virions that are then potentially available for *trans* infection of permissive target cells.

## Materials and Methods

### Viruses and cells

Three HIV-1 clade B isolates, 90US_873 (CCR5), 91US_4 (CCR5), and 89BZ_167 (CXCR4) and one clade A virus, 99KE_KNH1135 (CCR5) were isolated, propagated, and titrated in peripheral blood mononuclear cells (PBMC) obtained by leukapheresis, and stored in cell culture supernatant containing RPMI with 15% heat-inactivated fetal bovine serum as previously described [40]. Cells collected by leukapheresis were obtained under a protocol approved by the Institutional Review Boards of the Walter Reed Army Institute of Research and the Walter Reed Army Medical Center, and participants signed an informed consent document. Erythrocytes separated as a centrifuged pellet after Ficoll-Hypaque purification of PBMC were washed three times in Dulbecco's phosphate saline (lacking Ca^2+^ and Mg^2+^) (PBS) and stored in storage medium (1.1% dextrose monohydrate; 0.588% sodium citrate dihydrate; 0.41% NaCl; 0.276% monobasic sodium phosphate monohydrate; 0.042% citric acid monohydrate; 0.03% adenine) at 4°C for a maximum of three weeks. Before use, erythrocytes were quantified by microscopic counting in a hemocytometer; and were then washed with phenol red-free RPMI (lacking fetal bovine serum). To prepare ghosts, packed erythrocytes were hemolyzed in 10 volumes of ice-cold 5mM Tris-HCl/0.15 mM CaCl_2_/0.5 mM MgCl_2_, pH 8, and a small number of contaminating non-hemolyzed cells (leukocytes) were isolated or removed by centrifugation at 469×g at 4°C in a Sorvall RT6000D centrifuge with a H1000B rotor. Following removal of the leukocytes, the ghosts were pelleted by centrifugation at 20,000×g for 15 min at 4°C in a Sorvall RC-5B centrifuge with a SA-600 rotor. TZM-bl cells were grown as described [41].

### Binding of HIV-1 to cells

HIV-1 isolates, containing the indicated amounts of p24, were mixed 1∶1 (v/v) with the indicated number of erythrocytes (except as indicated in Fig. 1a), incubated at 4°C, with rotation (10 rpm), for 2 hr, washed 3 times with ice-cold phenol red-free RPMI medium by centrifugation at 469×g for 10 min. Bound p24 was measured by a p24 antigen capture ELISA (Advanced Bioscience Laboratories, Kensington, MD). Where indicated (Fig. 2A), erythrocytes were treated with 5 mM EDTA solution (Na salt) (Sigma-Aldrich, St Louis, MO) or in plastic 3 ml whole blood Vacutainer® tubes containing spray-coated EDTA (K salt) (BD, Franklin Lakes, NJ) for 10 min at 4°C. The erythrocytes were then centrifuged at 469×g for 10 min, and washed once in ice-cold phenol red-free RPMI medium. In a separate experiment (Fig. 2B) erythrocyte-bound HIV-1 was incubated with different amounts of EDTA (or no EDTA), and washed either in the absence or presence of Ca^2+^ and Mg^2+^. Where indicated (Figure 4), after binding the virus to erythrocytes or PHA-stimulated PBMC at 4°C, samples were incubated at either 4°C or 37°C for 4 hours. Then both erythrocytes and PBMC were treated with 5 mM EDTA (Na salt) for 10 min. The cells were washed with RPMI, the pellet was lysed, and p24 was measured.

### Infection of target cells

Infection of PBMC with free virus was performed as described [40]. For infection studies, erythrocytes were incubated with HIV-1 as described above, but the last wash was performed in, and resuspended in, 200 µl of RPMI with 15% heat-inactivated fetal bovine serum [40]. Samples were prepared in duplicate. Fifty µl of PHA-stimulated PBMC were then added at 1.5×10^5^ cells per well in 96-well deep-well round-bottom plates. After 4 days, 50 µl of culture fluid were harvested and assayed for p24 (measured by p24 antigen capture ELISA). For infection of CD4(+) TZM-bl cells with HIV-1, either 100 µl of free or erythrocyte-bound viral isolate was incubated with a 50 µl aliquot of 1×10^4^ TZM-bl cells (in Dulbecco's Modified Eagle's Medium with 15% heat-inactivated fetal bovine serum) in duplicate in 96-well flat-bottom culture plates. After 48 h, cells were lysed and luminescence was measured [41].
